# Synergistic Effects of NOTCH/γ-Secretase Inhibition and Standard of Care Treatment Modalities in Non-small Cell Lung Cancer Cells

**DOI:** 10.3389/fonc.2018.00460

**Published:** 2018-11-07

**Authors:** Venus Sosa Iglesias, Jan Theys, Arjan J. Groot, Lydie M. O. Barbeau, Alyssa Lemmens, Ala Yaromina, Mario Losen, Ruud Houben, Ludwig Dubois, Marc Vooijs

**Affiliations:** ^1^Department of Radiotherapy (MAASTRO), GROW-School of Oncology and Developmental Biology, Maastricht University Medical Center, Maastricht, Netherlands; ^2^Department of Psychology and Neuropsychology, MHeNS, Maastricht University Medical Center, Maastricht, Netherlands; ^3^MAASTRO Clinic, Maastricht, Netherlands

**Keywords:** non-small cell lung cancer, chemotherapy, targeted agents, radiation, NOTCH/gamma-secretase inhibitor, multicellular tumor spheroids

## Abstract

**Background:** Lung cancer is the leading cause of cancer death worldwide. More effective treatments are needed to increase durable responses and prolong patient survival. Standard of care treatment for patients with non-operable stage III-IV NSCLC is concurrent chemotherapy and radiation. An activated NOTCH signaling pathway is associated with poor outcome and treatment resistance in non-small cell lung cancer (NSCLC). NOTCH/γ-secretase inhibitors have been effective in controlling tumor growth in preclinical models but the therapeutic benefit of these inhibitors as monotherapy in patients has been limited so far. Because NOTCH signaling has been implicated in treatment resistance, we hypothesized that by combining NOTCH inhibitors with chemotherapy and radiotherapy this could result in an increased therapeutic effect. A direct comparison of the effects of NOTCH inhibition when combined with current treatment combinations for NSCLC is lacking.

**Methods:** Using monolayer growth assays, we screened 101 FDA-approved drugs from the Cancer Therapy Evaluation Program alone, or combined with radiation, in the H1299 and H460 NSCLC cell lines to identify potent treatment interactions. Subsequently, using multicellular three-dimensional tumor spheroid assays, we tested a selection of drugs used in clinical practice for NSCLC patients, and combined these with a small molecule inhibitor, currently being tested in clinical trials, of the NOTCH pathway (BMS-906024) alone, or in combination with radiation, and measured specific spheroid growth delay (SSGD). Statistical significance was determined by one-way ANOVA with *post-hoc* Bonferroni correction, and synergism was assessed using two-way ANOVA.

**Results:** Monolayer assays in H1299 and H460 suggest that 21 vs. 5% were synergistic, and 17 vs. 11% were additive chemoradiation interactions, respectively. In H1299 tumor spheroids, significant SSGD was obtained for cisplatin, etoposide, and crizotinib, which increased significantly after the addition of the NOTCH inhibitor BMS-906024 (but not for paclitaxel and pemetrexed), and especially in triple combination with radiation. Synergistic interactions were observed when BMS-906024 was combined with chemoradiation (cisplatin, paclitaxel, docetaxel, and crizotinib). Similar results were observed for H460 spheroids using paclitaxel or crizotinib in dual combination treatment with NOTCH inhibition and triple with radiation.

**Conclusions:** Our findings point to novel synergistic combinations of NOTCH inhibition and chemoradiation that should be tested in NSCLC *in vivo* models for their ability to achieve an improved therapeutic ratio.

## Introduction

More than two thirds of lung cancer patients are diagnosed at an advance stage (IIIb–IV). The lack of early diagnosis techniques together with intrinsic and acquired treatment resistance are obstacles for obtaining a cure, making lung cancer the deadliest type of cancer worldwide. Non-small cell lung cancer (NSCLC) accounts for 85–90% of lung cancers. Standard first line treatment for inoperable locally advanced stage IIIb NSCLC cancer is chemotherapy alone, or concurrent polychemotherapy with fractionated radiotherapy (RT). The latter, improved survival and locoregional control compared to sequential chemoradiation (1). Chemotherapy often involves the combination of two or more agents: one platinum-based (e.g., cisplatin, carboplatin) and one with a different mechanism of action (e.g., etoposide, paclitaxel, docetaxel, pemetrexed, vinorelbine, vinblastine). Targeted therapy is also an option when patients present oncogenic driver mutations (2). Despite the existence of diverse treatment options, median survival for stage IV NSCLC is only 8–10 months. This emphasizes the necessity to identify new treatment options for these patients.

Treatment selection is based on stage, the presence of driver oncogenic mutations, and general health status. More than 50% of Caucasian patients with lung adenocarcinomas, the most common NSCLC subtype, have unknown genetic alterations, and 25% of them bear mutations in the Kirsten Rat Sarcoma oncogene (*KRAS*) for which no targeted therapy is yet approved. The remaining patients bear driver mutations for which targeted therapies exists [e.g., *BRAF* (B-Rapidly accelerated fibrosarcoma serine/threonine protein kinase) mutations at 5% incidence, *RET* (Rearranged during transfection receptor tyrosine kinase) rearrangements at 2%, *MET* (proto-oncogene receptor tyrosine kinase also called hepatocyte growth factor receptor) amplifications at 5%] (2). However, there are Food and Drug Administration (FDA)-approved clinical grade targeted therapies for only three types of oncogenic driver mutations/rearrangements: mutations in the epidermal growth factor receptor (*EGFR*) with 10–12% incidence, the translocation in the anaplastic lymphoma kinase (*EML4-ALK*) with <5% incidence, and mutations in the receptor tyrosine kinase from the insulin family *ROS1* (v-ros UR2 sarcoma virus oncogene homolog 1 receptor tyrosine kinase) with <2% incidence. The FDA-approved tyrosine kinase inhibitors for these mutations include: erlotinib, gefitinib, afatinib (EGFR); ceritinib, alectinib (ALK); and crizotinib (ALK/ROS1) (2). However, the benefits for patients as measured by progression-free and overall survivals are modest and treatment is costly. An emerging obstacle in targeted therapeutics that hinders durable responses is acquired on-target resistance due to for example: 1) mutations in the driver oncogene, such as *EGFR* T790M (3), or *EML4-ALK* C1156Y or L1196M (4) thus causing resistance to first-line treatments, 2) activation of an alternative signaling pathway, such as *PI3K/AKT* (Phosphoinositide 3-kinase/Akt serine/threonine protein kinase also known as protein kinase B) for cisplatin resistance (5), and 3) histological transformation into small cell lung cancer (6), or epithelial-mesenchymal transition (7), among others.

The NOTCH signaling pathway is a highly conserved short-range cell-cell communication pathway important for organ development and tissue homeostasis in vertebrates (8). NOTCH signaling is crucial for normal lung organogenesis where it regulates broncho-alveolar and neuroendocrine cell differentiation (9). Activating mutations in *NOTCH1* or loss of *NUMB1*, a negative regulator of NOTCH, occur in 10–30% of NSCLC patients (10), except in the squamous cell carcinoma subtype, where *NOTCH* mutations are usually inactivating and function as tumor suppressors (11). High NOTCH activity has been associated with worse disease-free survival in patients, and increased proliferation, greater hypoxic fraction, and radioresistance in NSCLC tumor-bearing mice (12–14). NOTCH signaling has been shown to directly impact on the DNA damage response (15). Active NOTCH signaling has been linked, in different types of cancer, to resistance towards a broad range of chemotherapeutics and targeted agents, reviewed by Takebe et al. (16), including cisplatin (17), docetaxel (18), paclitaxel (19), gefitinib (20), anti-HER2 (human epidermal growth factor receptor 2) (21), anti-estrogens (22), dasatinib (23), temozolomide (24), doxorubicin and melphalan (25). Chemotherapeutic resistance is linked to high NOTCH signaling in lung cancer stem cells and worse outcome (26).

NOTCH receptors are transmembrane receptors that interact with membrane-bound ligands on adjacent cells. The rate-limiting step in NOTCH receptor activation is the intramembranous cleavage by the multi-enzyme complex γ-secretase, which enables nuclear translocation of the NOTCH intracellular domain, and target gene activation (27). Small molecule inhibitors against γ-secretase are potent inhibitors of NOTCH receptor activation under investigation in clinical trials (28) both as single agents, and in combination with chemotherapy, including: docetaxel in breast cancer (29), cisplatin in NSCLC (17), and other chemotherapeutics in different types of hematological and solid tumors (16), but also in combination with radiation in NSCLC (14). BMS-906024, a potent pan-NOTCH GSI in preclinical models (30), is currently undergoing clinical evaluation to determine safety and toxicity in patients with various hematological and solid tumors (NCT01292655, NCT01363817, and NCT01653470). Most lung cancer patients today receive combinations of chemotherapy and radiation as part of their standard treatment. While there is mounting evidence for a role of NOTCH signaling in resistance to chemotherapy and radiation alone, no study to date has investigated the effect of NOTCH signaling on combinations of radiotherapy and chemotherapy in NSCLC.

In this study, we therefore investigated whether a pan-NOTCH inhibitor, BMS-906024, enhances the current chemotherapy and radiotherapy single, and combination treatments, for NSCLC patients using 2D and 3D cell line models.

## Materials and methods

Refer to Table S5 for catalog numbers of reagents.

### Mammalian cell culture

The human non-squamous NSCLC cell lines NCI-H1299 (ATCC^®^ number: CRL-5803™) and NCI-H460 (ATCC^®^ number: HBT-177™) were cultured in DMEM and RPMI 1640 (Westburg), respectively for 2D assays and in DMEM for all 3D assays, all supplemented with 10% fetal calf serum (Sigma-Aldrich). According to the Cancer Cell Line Encyclopedia and the American Type Culture Collection, the H1299 cell line bears several mutations including partial deletion of TP53 (tumor protein 53), missense mutation NRAS^Q61K^ (N-Rapidly Accelerated Fibrosarcoma), and ALK (Anaplastic Lymphoma Receptor Tyrosine Kinase) hyperphosphorylation, whereas H460 bears mutations in MYC (V-Myc Avian Myelocytomatosis Viral Oncogene), ALK hyperphosphorylation, missense mutation KRAS^Q61H^, and PI3KCA (Phosphoinositide-3-kinase, catalytic, alpha polypeptide). The human osteosarcoma U2OS cells were cultured in DMEM supplemented with 10% fetal calf serum. U2OS-Jagged 2 overexpressing cells were kept under puromycin (2 μg/ml; BioConnect) selection as previously described (31). Cell line identities were confirmed by STR (Short tandem repeat) analysis (Identicell, Denmark) and they were all free of mycoplasma.

### Chemicals and drugs

The GSIs dibenzazepine (DBZ) and BMS-906024, were obtained from Syncom (the Netherlands) and Bristol-Myers Squibb (BMS), respectively. The National Cancer Institute/Cancer Therapy Evaluation Program provided a library consisting of 101 clinically approved anticancer agents, and was prepared as a 100 × stock in DMSO (77 μM). Cisplatin, etoposide, paclitaxel, docetaxel and pemetrexed were obtained from the hospital pharmacy. Crizotinib was obtained from Selleckchem. Dimethyl sulfoxide (DMSO) was purchased from Sigma-Aldrich.

### 2D proliferation assay

H1299 cells were seeded in 96 well Greiner plates (1,000 cells/well) and were incubated with 0.77 μM anticancer compounds 24 h after seeding, as single treatment or in combination with 2 or 4 Gy irradiation (Philips X-ray tube; 225 kV; 10 mA; dose rate of 1 Gy / 1.5 min), delivered 4 h after adding the compound. Where indicated, BMS-906024 (1 μM) was added to the combinations concomitantly with the chemotherapy regimen. Each treatment was assessed using at least 6–12 replicates per condition and was not refreshed throughout the course of the experiment (7 days). Cell confluency was monitored every 2–4 h using the IncuCyte™ FLR 2011A (32) in phase-contrast mode.

### Luciferase assays

H460 or H1299 cells were transfected in 6-well plates with a mixture of the NOTCH transcriptional reporter containing twelve consecutive RBP-Jκ/CSL binding sites driving Firefly luciferase (p12xCSL-FLuc), and a Renilla–luciferase expressing transfection control (pTK-RLuc) using polyethylenimine (P-PEI) (31). 8 h post transfection, cells were 6-fold diluted in 12-well Greiner plates, co-cultured with U2OS-Jagged 2 cells, and treated (16 h) with DBZ (0.2 μM) or BMS-906024 (1 μM) before measuring Firefly and Renilla luciferases (Promega). Transfection efficiency was normalized using Renilla luciferase. The normalized values of each condition were further normalized to control condition (DMSO ≥ 0.1%).

### Western blotting

H460 and H1299 cells were co-cultured with U2OS or U2OS-Jagged 2 expressing cells and treated (20 h) with BMS-906024 (0.1 or 1 μM) before protein extraction using RIPA buffer (50 mM TrisHCl, 150 mM NaCl, 1% Nonidet™ P-40, 0.5% sodium deoxycholate, 0.1% SDS, 100 mM sodium Fluoride, 2 mM sodium orthovanadate and supplemented with complete protease inhibitor cocktail). Protein concentrations were determined with Bradford Protein Assay (Bio-Rad). Proteins (60 μg) were separated on a 7.5% SDS-PAGE and transferred to PVDF membranes (VWR). Membranes were blocked with 5% non-fat dry bovine milk (Marvel) in PBS plus 0.05% Tween-20, subsequently incubated (O/N, 4°C) with rabbit monoclonal anti-NOTCH1 S3-Val1744 (1:1,000, Bioke) or rabbit anti-Lamin A (C-terminal) (1:1,000, Sigma-Aldrich), and visualized using HRP-linked secondary antibody (goat anti-rabbit, 1:2,500, Bioke) and Amersham ECL™ prime detection reagent (Sigma-Aldrich).

### 3D multicellular tumor spheroid (MCTS) assay

H1299 cells (1,000 cells/well) were seeded in a 96-multiwell Greiner plate coated with 50 μl autoclaved 1.5% agarose (Sigma-Aldrich) in serum-free DMEM medium to allow multicellular spheroid formation as previously described (33). H460 cells (500 cells/well) were seeded in 96-multiwell Ultra-Low attachment plates (BioScience) as described by manufacturer (32). On the fourth day post seeding, one individual spheroid with an average volume of 0.063 μm^3^ for H1299 and 0.087 μm^3^ for H460 was formed/well and reference phase-contrast images were acquired for each spheroid. Subsequently, a minimum of 12 spheroids/condition were treated with the following regimens: 72 h anticancer agent (cisplatin, etoposide, paclitaxel, docetaxel, pemetrexed or crizotinib) or vehicle alone or combined with concomitant 7-days BMS-906024/vehicle. Drug vehicles were either medium or DMSO ≥ 0.05%. Additionally, a single radiation dose (0, 2 or 4 Gy; Philips X-ray tube; 225 kV; 10 mA; dose rate of 1 Gy / 1.5 min) was delivered alone, or in combination with the aforementioned drugs. BMS-906024 was refreshed after 72 h of treatment. Drug treatment washout (50% replacement with untreated complete DMEM medium) was performed 72 h post anticancer drug treatment, 7 days post BMS-906024 treatment, and continued 3 × /week until endpoint. Phase contrast images were acquired (Olympus IX81 inverted microscope) up to 3 × /week and analyzed using SpheroidSizer (34), a MATLAB (R2016a)-based open-source software to assess spheroid volume using an active contour algorithm. Spheroid growth was monitored until their volume reached at least up to 4 × (but on average above 10–15 ×) the volume at the start of treatment (T4xSV) depending on the severity of treatment. Spheroid specific spheroid growth delay (SSGD) was calculated as T4xSV_treatment_ – T4xSV_control_ and corrected for the doubling time of the control group.

### Statistical analysis

Statistical analyses were performed using GraphPad Prism Software (v5.03). Comparison between slope and y-intercept parameters of linear regression equations was used to assess the differences in the linear region (between 20 and 86% confluency) of the 2D growth rate of cancer cells (**Figure 2**). Normality distribution of 3D MCTS specific spheroid growth delay data was assessed with a D'Agostino and Pearson omnibus test and confirmed by Skewness / kurtosis test (*n* > 8). Significant outliers from 3D MCTS specific growth delay were determined with Grubb's test and excluded from the study. Comparisons between groups were performed using one-way ANOVA with Bonferroni *post-hoc* test to correct for multiple testing. A two-way ANOVA was applied to test the interaction (synergism) between grouped data of BMS-906024 and chemotherapy, RT, or chemoradiation. A *p*-value < 0.05 was considered statistically significant.

## Results

### Sensitivity of non-small cell lung cancer cells to drug-radiation combinations

**Figure 1:**
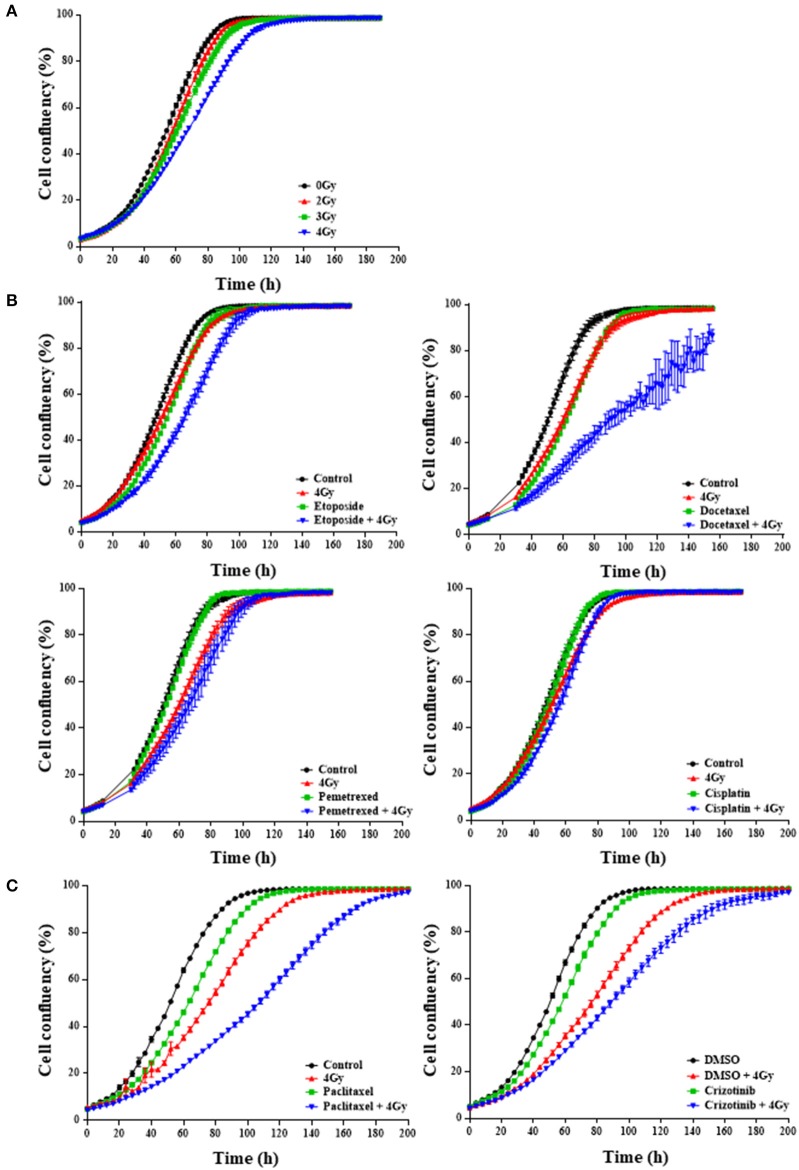
First-line chemotherapeutics combined with radiation delays H1299 monolayer growth. IncuCyte™ FLR analysis of cell confluency (%) over time for NCI-H1299 cells growing in monolayer treated with increasing RT doses between 2 and 4 Gy **(A)** and the selected RT dose of 4 Gy in combination with/without chemotherapeutic agent at 0.77 microM **(B)**, 2.5 nM for paclitaxel (**C**, left) or 0.4 microM for crizotinib (**C**, right). “Control” indicates the use of medium. Alternatively, DMSO was used where indicated. A minimum of two independent experiments and 6–18 wells/condition/experiment were tested. Mean and standard error of the mean are plotted.

In order to identify drugs that interact with radiation, we screened 101 FDA-approved anti-cancer drugs from the CTEP library in two different NSCLC cell lines: H1299 and H460. We first optimized the radiation dose and determined that a single radiation dose of 4 Gy resulted in a statistically significant (*p* < 0.0001) proliferative delay as compared to non-irradiated cells (Figure 1A). We then assessed whether a single dose per compound with 4 Gy affected NSCLC cell growth (Figures 1B,C). IncuCyte growth analysis suggested that 21% of the drug-radiation interactions between compounds and RT were synergistic, 17% were additive, 7% were protective and 27% had no effect (Figures 1B,C; Tables S1.1, S1.2). For 29% of the compounds, the dose of 0.77 μM was already too toxic in the absence of RT. Additionally, the CTEP library was screened in the H460 NSCLC cell line and graphical analysis revealed that 5% of the drug-radiation interactions were synergistic, 11% were additive, 14% were protective and 64% had no effect when combined with 4 Gy radiation. For 15% of the compounds, the dose selected was too toxic (Tables S2.1, S2.2). Some of the compounds that enhanced (synergistically or additively) growth delay in combination with RT in both cell lines include irinotecan, mitotane, and dasatinib which are not part of the standard of care for NSCLC. We focused further on those compounds that are commonly used as a part of the first-line treatments in NSCLC. We observed in H1299 cells that docetaxel, etoposide, pemetrexed, and paclitaxel, all synergistically interacted with RT (Figures 1B,C) and that crizotinib only showed an additive interaction when combined with RT (Figure 1C). At the dose tested, cisplatin did not show any effect as monotherapy nor when used in combination with RT (Figure 1B). The effects observed in H460 cells were similar for etoposide and crizotinib which interacted synergistically with RT.

### NOTCH inhibition when combined with chemotherapy and chemoradiation reduces 2D cell proliferation

**Figure 2:**
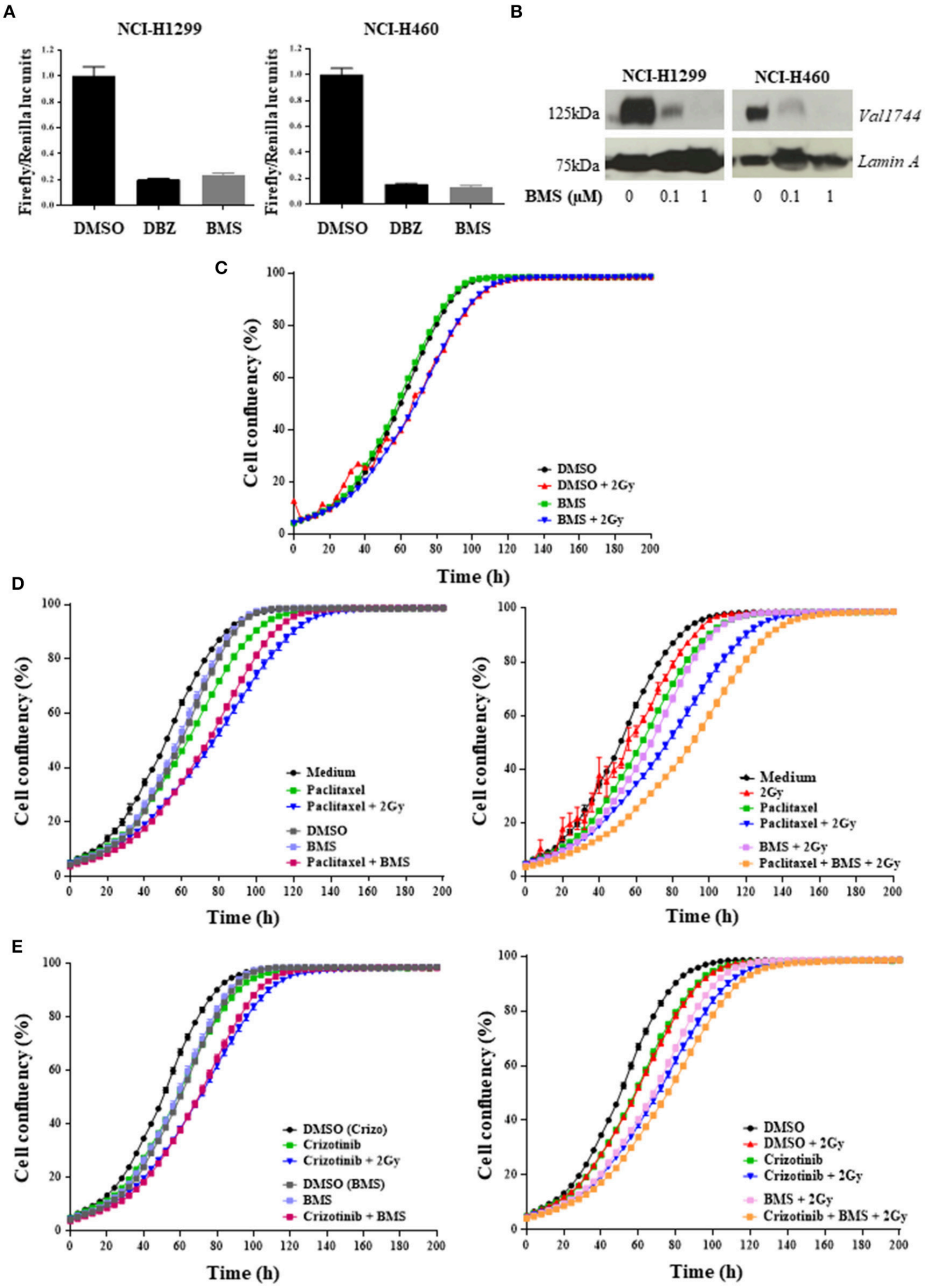
Pan-NOTCH/γ-secretase inhibitor BMS-906024 delays cell proliferation of H1299 cells treated with chemoradiation. Luciferase reporter assay **(A)** and Western Blot **(B)** of NCI-H1299 and NCI-H460 treated with DMSO (vehicle) or either 0.2 μM dibenzazepine (DBZ), or 0.1 or 1 μM BMS-906024. Incucyte analysis of cell confluency (%) over time of NCI-H1299 cell line for BMS-906024 1μM with/without 2 Gy radiation **(C)**; 2.5 nM paclitaxel **(D)** or 0.4 μM crizotinib **(E)** compared to dual treatment including 1 μM BMS-906024 (left) or comparing chemoradiation with 2 Gy to triple combination including 1 μM BMS-906024 (right). A minimum of two independent experiments and 6–18 wells/condition/experiment were tested. Mean and standard error of the mean are plotted.

To assess whether H1299 and H460 NSCLC cells lines have active NOTCH signaling, we measured NOTCH transcriptional activity using a 12xCSL-luciferase based reporter assay. NOTCH transcriptional activity, when in co-culture with U2OS-JAGGED 2 expressing cells, was blocked after treatment with γ-secretase inhibitors: BMS-906024 or dibenzazepine (DBZ) (Figure 2A). BMS-906024 also blocked NOTCH1 cleavage of H1299 and H460 cells, in a dose-dependent manner, as determined by immunoblot using antibodies recognizing the activated Val1744-cleaved NOTCH1 intracellular domain (NICD1) (Figure 2B). BMS-906024 however, did not affect the 2D monolayer growth of H1299 cells alone nor when combined with 4 Gy radiation (Figure 2C).

Next, we investigated whether NOTCH inhibition altered the response of H1299 cells treated with chemotherapeutics. For this, we selected two agents with different mechanisms of action: the anti-mitotic taxane paclitaxel, and the ALK/ROS1/c-MET small molecule inhibitor crizotinib, and evaluated the effect of NOTCH inhibition both with and without RT. Both anticancer agents induced a significant (*p* < 0.0001) inhibition of proliferation when combined with BMS-906024. The chemotherapy plus GSI effect was similar to the effect observed for the chemoradiation (2 Gy) (Figures 2D,E). This significative decrease in confluency was also detected when BMS-906024 was added to chemoradiation (2 Gy) with paclitaxel (*p* < 0.0001) both at 2.5 nM (Figure 2D) and 1 nM (data not shown), but not for crizotinib (Figure 2E).

### NOTCH inhibition enhances specific growth delay in 3D tumor spheroids combined with chemotherapy and chemoradiation

**Figure 3:**
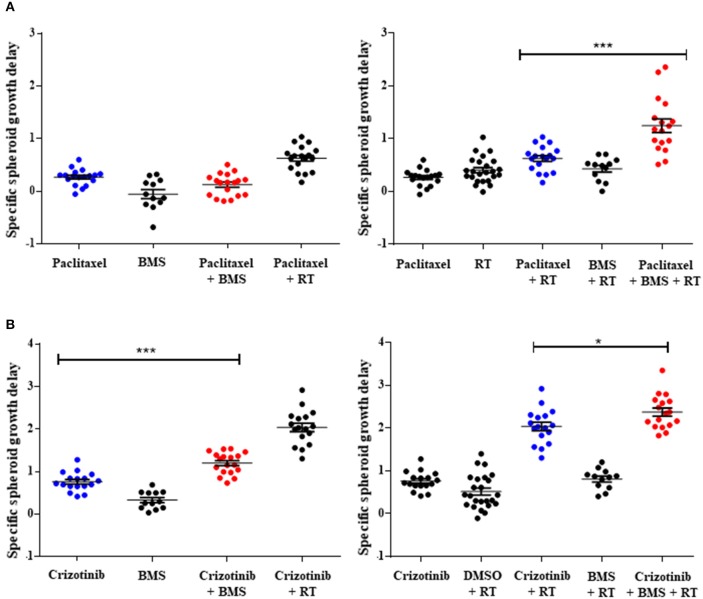
Pan-NOTCH/γ-secretase inhibitor BMS-906024 delays H1299 multicellular spheroid specific growth in combination treatments. SSGD of multicellular NCI-H1299 spheroids were treated with 2.5 nM paclitaxel **(A)** or 0.4 μM crizotinib **(B)** and/or 1 μM BMS-906024 and/or 2 Gy radiation, is shown. Statistical significance of the comparison between single-agent chemotherapy: paclitaxel **(A)** or crizotinib **(B)**, vs. chemotherapy plus BMS-906024 (left), or chemoradiation vs. chemoradiation plus BMS-906024 (right) is shown. Spheroid specific spheroid growth delay (SSGD) was calculated as T4xSV_treatment_ – T4xSV_control_ and corrected for the doubling time of the control group. A minimum of three independent biological replicates with 12 spheroids/condition/experiment were tested. Mean and standard error of the mean are plotted. *p*-value < 0.001 (^***^) and < 0.050 (^*^).

While 2D drug screening platforms are amendable to fast high-throughput assays, it is well established that chemotherapy and radiation responses are more representative in 3D multicellular tumor spheroids (MCTS). We therefore sought to evaluate whether NOTCH targeting with BMS-906024 would enhance synergistically chemotherapy or chemoradiotherapy treatments in 3D spheroid cultures. We first established that NOTCH signaling was active in multicellular tumor spheroids of H1299 and H460 cells, where we show that NOTCH target genes *HES1* and *cMYC* were expressed and their expression could be blocked with 1 μM BMS-906024 already 2-days post-treatment (Figure S1). Next, we assessed the specific spheroid growth delay (SSGD) for monotherapy treatments in both H1299 and H460 MCTS. BMS-906024 (1 μM) monotherapy had a significant (*p* < 0.05) specific spheroid growth delay (SSGD), as well as single chemotherapy (paclitaxel, crizotinib, *p* < 0.0001), and single dose RT (2 or 4 Gy; Figure 3; data not shown). In addition, when BMS-906024 (0.1 or 1 μM) was combined with crizotinib in both H1299 and H460 MCTS, SSGD increased significantly (*p* < 0.05; Figure 3, Figure S1; Table S4). This was not the case for paclitaxel when combined solely with BMS-906024. Moreover, in H1299 MCTS, BMS-906024 combined with chemotherapeutics like cisplatin, and etoposide, for at least one concentration tested, increased SSGD (Table 1, Figure S3).

**Table 1:** Synergistic interactions and statistical significance between combination treatments with and without BMS-906024 on SSGD.

	**Chemo vs. Chemo** + **BMS-906024**		**Chemo vs. ChemoRT** + **BMS-906024**	
	**SSGD**	**Synergism**	**SSGD**	**Synergism**
**H1299**				
Cisplatin^ɤ^	^***^	ns	^*^	^**^
Etoposide	^***^	ns	^**^	ns (0.07)
Paclitaxel	ns	ns	^***^	^***^
Docetaxel	^***^(BMS effect)	^*^	^***^	^*^
Pemetrexed^ɤ^	ns	ns	ns	ns
Crizotinib	^**^	ns	^*^	^***^
**H460**				
Paclitaxel	ns	ns	^***^	^***^
Crizotinib	^***^	^***^	^***^	^***^

Chemotherapeutics (1.2 μM Cisplatin, 0.1 μM Etoposide, 2.5 nM Paclitaxel, 1 nM Docetaxel, 0.25 μM Pemetrexed, 0.4 μM (H1299) or 0.8 μM (H460) Crizotinib) were combined with 1 or 0.1 μM (^ɤ^) of γ-secretase inhibitor BMS-906024 and/or single dose 2 Gy radiation, and compared to their respective treatment control without BMS-906024. A minimum of 12 individual spheroids were tested per condition. Ns, not significant;

*** *p-value < 0.001*,

** p-value < 0.01 and

* *< 0.050. “BMS effect” note indicates that there was no difference between BMS-906024 treatment and the combination treatment of BMS-906024 with chemotherapeutic*.

Combination of 1 μM BMS-906024 with single dose RT (2 Gy) also induced a SSGD (*p* < 0.05; Figure 3; Tables 1, Figure S3). Chemoradiation (paclitaxel or crizotinib plus 2 Gy) increased SSGD significantly (*p* < 0.001) compared to either monotherapy. Moreover, the addition of BMS-906024 (0.1 μM or 1 μM) to chemoradiation (both with paclitaxel, crizotinib), further enhanced (*p* < 0.05) SSGD in both H1299 and H460 MCTS (Figure 3; Table 1; Figure S2; Tables S3, S4). Similar effects were found when we added, in H1299 MCTS, BMS-906024 to chemoradiation regimens using the other chemotherapeutics tested (cisplatin, etoposide, and docetaxel; Table 1, Table S3).

Clonogenic survival assays performed on disaggregated MCTS treated with paclitaxel, BMS-906024 and/or RT, whilst in 3D-MCTS format, suggested that the triple therapy combination was more effective in blocking clonogenic survival than paclitaxel, and RT alone or in combination (Figure S3).

Importantly, we observed synergistic interactions in H1299 MCTS between cisplatin, etoposide, docetaxel, or crizotinib (also in H460 MCTS; Table S4) and BMS-906024 (Tables 1, Table S3). Moreover, with the exception of pemetrexed and etoposide, we observed synergistic interactions when BMS-906024 was added to chemoradiation (Table S3). Additionally, the interaction between at least one concentration of both chemotherapies tested (paclitaxel, crizotinib) plus radiation with BMS-906024 in H460 MCTS, was synergistic (Table S4).

### Top-ranking treatment selection between treatment modalities differs

**Figure 4:**
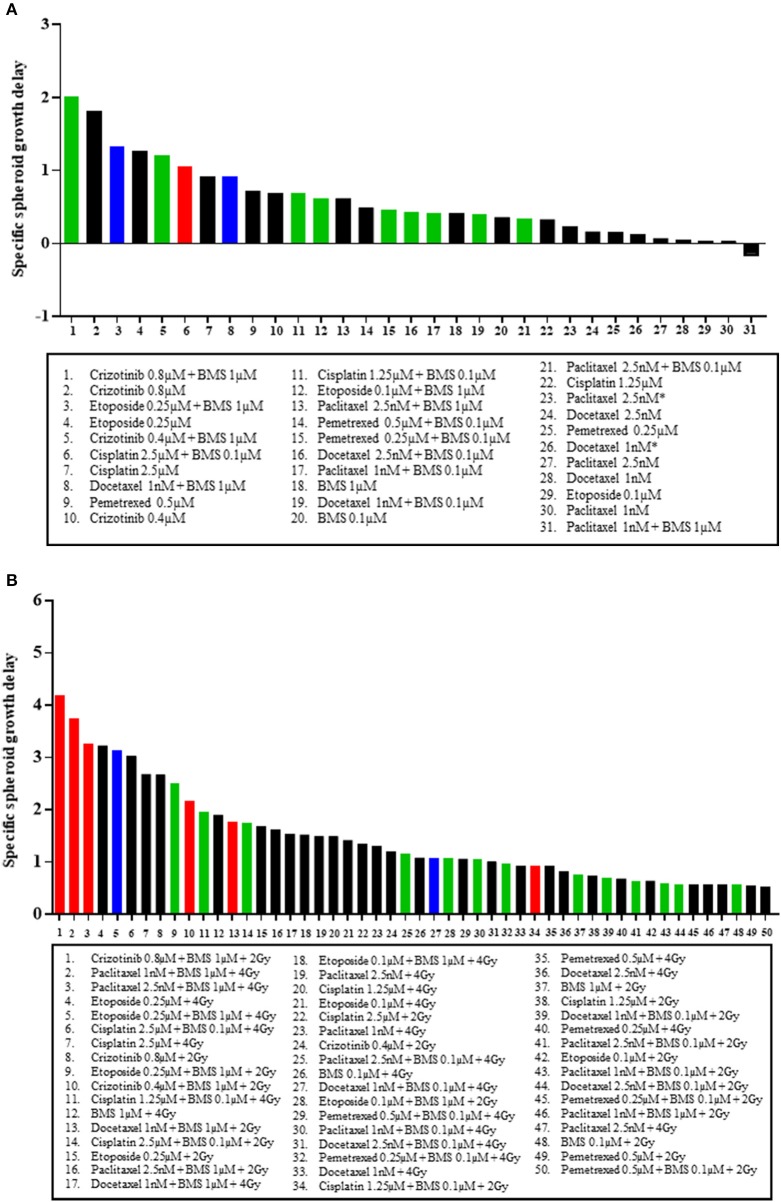
Top ranking treatment combinations in NSCLC multicellular spheroids. Comparisons in multicellular NCI-H1299 spheroids of: **(A)** chemotherapeutic at dose specified, and chemotherapeutic plus 0.1 or 1 μM BMS-906024; and **(B)** chemoradiation (with 2 or 4 Gy), and chemoradiation plus 0.1 or 1 μM BMS-906024. Red bars indicate that the addition of BMS-906024 to that treatment option conferred a statistically significant SSGD compared to the treatment without BMS-906024, and the interaction was synergistic. Green bars indicate only statistically significant SSGD. Blue bars indicate only synergistic interaction. Black bars indicate non-significant relationship.

Finally, we ranked, based on spheroid specific growth delay, the therapeutic effects of all tested combinations from both H1299, and H460 MCTS models to answer two questions: which treatment modality gave the best response in terms of SSGD and synergistic interaction when comparing (1) chemotherapy vs. chemotherapy plus BMS-906024 (Figure 4A, Figure S4A), and (2) chemoradiation vs. chemoradiation plus BMS-906024 (Figure 4B, Figure S4B). Top-ranking treatments involve combinations with NOTCH inhibition. In the H1299 MCTS model, we observed the strongest synergistic interactions between either crizotinib (0.8 μM) or etoposide (0.25 μM) with BMS-906024 (Figure 4A). Similarly, in the H460 model, crizotinib (0.8 μM) with BMS-906024 was the top-ranking interaction in terms of both statistical significance in SSGD and synergism (Figure S4A). When comparing chemoradiation with and without NOTCH inhibition in the H1299 model, the top-ranking interactions based on both SSGD statistical significance and synergism are crizotinib (0.8 μM) followed by paclitaxel, both in triple combination (Figure 4B). Similarly, in the H460 model, crizotinib (0.8 μM) in triple combination was the best (Figure S4B).

## Discussion

In this study, we sought to identify synergistic interactions between a NOTCH inhibitor and chemotherapy alone or chemotherapy in combination with radiation. Our results in 2D suggest that the effect of first-line chemotherapeutics in combination with radiation in non-squamous NSCLC treatment was diverse and limited by the selection of a dose of drug-radiation to perform the screen. Interestingly, some of the chemotherapeutics identified that showed sensitization with RT in both cell lines, are not used for NSCLC treatment (e.g., irinotecan, mitotane, dasatinib) in Europe/North America, although irinotecan seems to have a favorable effect on small-cell lung cancer in Japanese patients. This sets an opportunity for the use of new clinically approved drugs in NSCLC treatment to further look into understanding their underlying mechanisms.

In general, we observed fewer therapeutic interactions of chemotherapeutics in combination with radiotherapy in H460 compared to H1299 cells, which could be explained by a different mutation pattern. The H460 cell line expresses wildtype *TP53* mRNA levels whereas the H1299 cell line has a partial deletion of the *TP53* gene and lacks its expression. This could sensitize H1299 cells to combination treatments with other DNA damaging agents. However, recently it was demonstrated that the leucine zipper containing ARF-binding protein (LZAP), which binds and stabilizes TP53, is correlated with TP53 in human NSCLC. Knockdown of LZAP in cancer cells expressing wild-type TP53 protects them from DNA damage-induced cell death whereas cancer cells expressing mutant TP53 were sensitized to DNA damage by LZAP knockdown (35). The LZAP status in H460 has not been reported, but if it were deleted, it could explain why H460 cells with wild type TP53 are more resistant to treatment. Another study suggested that NOTCH1 inhibition induces TP53-dependent apoptosis as a consequence of increased TP53 stability, and that TP53 status has an impact on NOTCH1 signaling and lung tumorigenesis (36). However, the best responding cell line in our study, H1299, has a partial deletion of *TP53*, therefore, it seems that TP53 status is not always correlated with therapeutic outcome.

H1299 cells were reported to be intrinsically more cisplatin-resistant than H460 cells due to the inability to trigger an apoptotic response, possibly due to the lack of TP53 expression (37). Additionally, H1299 was categorized as a paclitaxel-resistant cell line as compared to four other epithelial-derived carcinoma types (nasopharyngeal, gastric, breast and hepatocellular) in terms of cellular apoptosis, mitochondrial functionality and colony-forming capacity (38). In our 2D IncuCyte proliferation assay we showed that paclitaxel-induced proliferation reduction, at a 40 × lower dose, was similar to the reduction seen using an MTT assay 72 h post-treatment as reported by Shen et al. (39).

A major limitation of 2D monolayer assays is that they do not recapitulate tumor physiology in terms of limited diffusion of oxygen (thus creation of a hypoxic niche), nutrients, metabolites, and signaling molecules; cell-cell interactions; proliferative index; differentiation; and sensitivity to radiation and chemotherapy as opposed to 3D multicellular tumor spheroids (MCTS). To overcome some of the limitations of 2D assays, we addressed the interaction of chemotherapeutics with NOTCH inhibition with and without radiation treatment in 3D MCTS assays, where we observed that for most of the tested compounds, addition of BMS-906024 to chemotherapy or chemoradiation regimens resulted in a greater specific spheroid growth delay. Interestingly, the compounds tested had different mechanisms of action, reflecting that NOTCH is able to crosstalk with several pathways. NOTCH receptor targeting may target indirectly several mechanisms of therapeutic resistance including: angiogenesis (40), hypoxia (41), EMT (42), and possibly autophagy (43).

NOTCH1 activation has been linked with poor prognosis in NSCLC, specifically in those patients without *TP53* tumor suppressor mutations (10), such as the H460 cell line. The strongest combination treatment in both spheroid tumor models was that which included crizotinib in combination with NOTCH inhibition and radiation. Interestingly, both cell lines have not been reported to carry the common ALK translocation. They do have however, increased phosphorylated Y1604 ALK expression shown to promote tumorigenesis through activation of the downstream targets: STAT3 (Signal transducer and activator of transcription 3), AKT, and ERK (Extracellular signal-regulated kinases), and predisposing tumors to crizotinib (44). Phase I clinical trials with crizotinib resulted in 61% of partial or complete responses. However, most patients develop resistance to crizotinib within 12 months due to *de novo* EML4-ALK mutations (C1156Y or L1196M), ALK gene amplification, or alternative mechanisms, such as epithelial-to-mesenchymal transition (EMT) or upregulation of P-glycoprotein (4). This has fueled development of 2^nd^ generation ALK inhibitors that target crizotinib-resistant ALK translocations; however, patients also develop resistance to the latter. Our findings suggest increased sensitization of NSCLC tumor spheroids when adding GSI to crizotinib alone or in combination with radiation. It will be interesting to test whether NOTCH inhibition is also able to overcome crizotinib resistance. Moreover, the enhanced effects of crizotinib were slightly higher in the H1299 model, which is KRAS wildtype. This is in line with a recent publication which concluded that BMS-906024 not only sensitizes NSCLC to paclitaxel, but that this occurs more potently in KRAS and BRAF wildtype cancers therefore, possibly being able to predict better patient outcomes to dual combination therapy (19). On the other hand, other studies report that canonical NOTCH pathway is needed for the tumorigenesis of KRAS^G12V^ driven NSCLC and that pharmacological inhibition with GSI arrests tumor growth partly via activation of DUSP1 and consequent dephosphorylation of ERK specifically (not MEK) (45).

It has been reported that cisplatin treatment enriches NOTCH1 and CD133-expressing lung cancer stem-like cells from H460 and H661, induce DNA damage responses, and upregulate ABC drug transporters which in turn, increases cross-resistance to other chemotherapeutics (doxorubicin and paclitaxel). GSI-IX DAPT (N-[N-(3,5-Difluorophenacetyl)-L-alanyl]-S-phenylglycine *t*-butyl ester) pre-treatment was able to reduce the number of CD133-expressing cells and sensitized tumor cells to doxorubicin and paclitaxel (17). This is complementary to our observation in this study where the triple combination of cisplatin, NOTCH inhibition and RT treatment synergistically enhances SSGD. Other studies have also investigated the effect of different NSCLC chemotherapeutics (e.g., cisplatin, vinorelbine, EGFR inhibitors, c-Met inhibitors) using 3D NSCLC MCTS models (46, 47). These studies mostly address short-term effects, up to 72 h, on proliferation and viability using drug concentrations several folds greater than those used in this study. A clinically more relevant parameter, used in this study, to assess treatment efficacy is long-term growth delay or “*in vitro* local control” thereby making our screening model relevant for follow-up research *in vivo*. Additionally, all chemotherapeutic doses used in this study are within clinical range (compared to plasma concentrations in clinical trials, Supplementary Information). However, it must be noted as well that the model used in this study has its limitations in recapitulating certain aspects of the *in vivo* tumor microenvironment that will impact response to chemotherapy (48) and radiotherapy (49). It has been shown that tumor cell interactions with the extracellular matrix interactions influence radiation sensitivity and chemotherapy response through activation of cell survival and DNA damage response pathways (50). In addition, it is well established that cancer-associated fibroblasts (CAF) contribute to tumor progression and treatment resistance through a variety of mechanisms including secretion of pro-survival factors for cancer stem cells (51). However, monoculture of MCTS compared to coculture with CAF or normal lung fibroblasts does not always yield differential survival outcomes (46) nor responses to treatment, and hence, other elements of the tumor microenvironment should be considered. The ideal model consisting of patient-derived: tumor cells, endothelial cells, CAFS, and immune cells, is yet to be optimized.

Several clinical studies using NOTCH/γ-secretase inhibitors have shown limited effect on local control and have been halted. One of the major concerns has been gastrointestinal (GI) toxicity caused by NOTCH inhibition using GSI in preclinical models (52). GI toxicity can be mitigated by glucocorticoid treatment (53) or via intermittent scheduling (24). Significantly, the synergistic interactions of some combination treatments including NOTCH inhibition in this study, suggest that lower doses of individual treatments may be used, thus having the potential to limit normal tissue toxicity at the same therapeutic efficacy. This would enable treatment prolongation which potentially could increase survival. However, because NOTCH blockade also alters tumor vasculature, combination treatments including radiation and chemotherapy require careful scheduling. In clinical practice, the use of concomitant polychemotherapy regimens with fractionated RT in locally advanced NSCLC has been reported to improve survival. Thus, follow-up studies should assess whether the chemotherapeutic plus NOTCH inhibition regimen is more efficacious in delaying tumor growth compared to the current polychemotherapy regimens, both with fractionated RT. The use of complementary predictive and companion biomarkers to stratify patients based on NOTCH expression would further increase the potential of NOTCH-based therapeutic strategies. Additionally, because a common (20–30% incidence) comorbidity in NSCLC patients is metastasis to the brain, which is enhanced (44–60% incidence) among patients with druggable oncogene drivers (EGFR, ALK) (54), it is of interest to investigate whether NOTCH inhibitors that cross the blood-brain barrier can prevent or reduce brain metastasis formation.

Altogether, NOTCH targeting is very attractive for further research in *in vivo* models. However, it should be noted that this effect was only tested in two cell lines. It would be interesting to extend these studies to a greater cell line panel, taking along all the different NSCLC subtypes with both a dependence and an independence of NOTCH signaling, to determine the subset of responding cell lines.

## Conclusions

The therapeutic benefits found in 2D growth assays in this study portray the wide variety of mechanisms of action of anti-cancer agents that are effective for NSCLC cells, and it brings up attractive possibilities of novel therapeutic combinations for NSCLC treatment. Because in this semi-high throughput 2D screen the anti-cancer agents are clinically approved, extrapolation of data to *in vivo* trials would be faster since maximum tolerated doses (MTD) in mice and patients are already known. In 3D multicellular NSCLC spheroids, the addition of the NOTCH inhibitor BMS-906024 to several chemotherapeutic agents with different mechanisms of action increased significantly specific spheroid growth delay and some, interacted synergistically. Our findings support a re-evaluation of the application of GSI in combination therapy for advanced non-squamous NSCLC and follow-up research in *in vivo* preclinical models.

## Availability of data and material

Data is presented as Supplementary Information, figures and tables. The SpheroidSizer MATLAB (R2016a)-based open-source software to assess spheroid volume can be accessed in Chen *et al*. (34).

## Author contributions

VSI carried out most of the experiments, analyzed and interpreted the data. JT, LMOB, AL, and AG assisted in some experiments. AY and ML assisted in automating image acquisition of spheroids. RH supervised statistics used in the study. JT, LD, and MV supervised the project. VSI, JT, LD, and MV wrote the paper. All authors read and approved the final manuscript.

### Conflict of interest statement

The authors declare that the research was conducted in the absence of any commercial or financial relationships that could be construed as a potential conflict of interest.
