# Supplementary material for: Synergistic Effects of NOTCH/γ-Secretase Inhibition and Standard of Care Treatment Modalities in Non-small Cell Lung Cancer Cells
Source: Front Oncol. 2018 Nov 7;8:460. doi: 10.3389/fonc.2018.00460 (PMC6234899; doi:10.3389/fonc.2018.00460)
Supplement: Supplementary file 1 [file Data_Sheet_1.pdf]

SUPPLEMENTARY INFORMATION

Clinical relevance of drug concentrations

The clinical relevance of the tested drug concentration may be assessed using the area under the plasma concentration-time curve (AUC). The AUC of NSCLC patients treated with:

1. 25 mg/m^2 (RP2D) cisplatin for non-elderly (20-74 years) patients is 91.8 ± 11.5 µg/ml x h and 94.3 ± 12.6 µg/ml x h for elderly (>75 years) patients delivered in infusions 1x/week for 3weeks [Minami, H. *et al.* 2004].
2. 360 mg/m^2 Etoposide (VP-16) for 72h-continuous venous infusion is 384 ± 88.4 mg/l/h or if repeated daily via iv administration is 360.6 ± 91.8 mg/l/h [Chatelut, E. *et al.* 1990]. 25 mg oral etoposide is 7.09 ± 1.26 or 25 mg delivered twice is 8.67 ± 0.77 µg*h/ml [Katoh, O. *et al.* 1991].
3. 60, 180, and 300 mg/m^2 (MTD) of oral paclitaxel are 1.65 ± 0.93, 3.33 ± 2.39, and 3.46 ± 1.37 µmol/L x h, respectively and for 175 mg/m^2 iv paclitaxel is 15.39 ± 3.26 µmol/L x h [Malingre, M.M. *et al.* 2000]. 200mg/m^2 iv paclitaxel is 16,657 ng*h/ml (geometric least square mean AUC) [Rodon, J. *et al.* 2012].
4. 35 mg/m^2 (recommended phase II dose, RP2D) docetaxel in non-elderly (20-74 years) patients is 1.4 ± 0.64 µg/ml x h and for 20 mg/m^2 (RP2D) docetaxel in elderly (>75 years) patients is 0.79 ± 0.34 µg/ml x h, both doses delivered in infusions 1x/week for 3weeks [Minami, H. *et al.* 2004].
5. Single dose of 250mg crizotinib is 1402 ng*h/ml (318.6 ng*h/ml for its metabolite PF-06260182) [Tan, W. *et al.* 2017].
6. 400, 500, 600, or 700 mg/m^2 pemetrexed infusion on day 1 of a 21-day cycle are 130 ± 36.4, 194 ± 75.8, 187 ± 30.5, and 216 ± 29.3 µg*h/ml respectively [Li, K.M. *et al.* 2007].

The conversion of these AUC doses to molarities (mol/L)*hour of exposure gives a reference of the concentrations that shouldn't be exceeded *in vitro*:

1. 305-314 µM*h for cisplatin infusion ($MW_{cisplatinl}$: 300.01 g/mol),
2. 652 µM*h for infusion and 613 µM*h for oral etoposide ($MW_{etoposide}$: 588.557 g/mol),
3. 3.46 µM*h for oral and 15.39-19.5 µM*h for iv paclitaxel ($MW_{paclitaxel}$: 850.906 g/mol),
4. 0.98-1.73 µM*h for docetaxel infusion ($MW_{docetaxel}$: 807.879 g/mol),
5. 3.11µM*h for Crizotinib ($MW_{crizotinib}$: 450.337 g/mol),
6. 304-505 µM*h for pemetrexed infusion ($MW_{pemetrexed}$: 427.411 g/mol),

When these values are corrected for exposure time (72h in multicellular tumor spheroid assays), the drug doses used in this study are all below the above reference limits.
Because the NOTCH inhibitor DBZ has not proceeded to clinical trials and the clinical trials involving the NOTCH inhibitor BMS-906024 are still in the recruiting phase, there are no pharmacokinetic reference limits established.

REFERENCES:

Chatelut, E., Chevreau, C., Blancy, E., Lequellec, A., Canal, P., Roche, H., et al. (1990). Pharmacokinetics and toxicity of two modalities of etoposide infusion in metastatic non-small-cell lung carcinoma. *Cancer Chemother Pharmacol* 26(5)**,** 365-368.

Katoh, O., Yamada, H., Hiura, K., Aoki, Y., and Kuroki, S. (1991). Clinical pharmacology and toxicity of low daily administration of oral etoposide in advanced lung cancer patients. *J Clin Pharmacol* 31(12)**,** 1155-1160.

Li, K.M., Rivory, L.P., and Clarke, S.J. (2007). Pemetrexed pharmacokinetics and pharmacodynamics in a phase I/II study of doublet chemotherapy with vinorelbine: implications for further optimisation of pemetrexed schedules. *Br J Cancer* 97(8)**,** 1071-1076. doi: 10.1038/sj.bjc.6603995.

Malingre, M.M., Terwogt, J.M., Beijnen, J.H., Rosing, H., Koopman, F.J., van Tellingen, O., et al. (2000). Phase I and pharmacokinetic study of oral paclitaxel. *J Clin Oncol* 18(12)**,** 2468-2475. doi: 10.1200/jco.2000.18.12.2468.

Minami, H., Ohe, Y., Niho, S., Goto, K., Ohmatsu, H., Kubota, K., et al. (2004). Comparison of pharmacokinetics and pharmacodynamics of docetaxel and Cisplatin in elderly and non-elderly patients: why is toxicity increased in elderly patients? *J Clin Oncol* 22(14)**,** 2901-2908. doi: 10.1200/jco.2004.10.163.

Rodon, J., Jacobs, C.D., Chu, Q., Rowinsky, E.K., Lopez-Anaya, A., Takimoto, C.H., et al. (2012). A phase I pharmacokinetic study of bexarotene with paclitaxel and carboplatin in patients with advanced non-small cell lung cancer (NSCLC). *Cancer Chemother Pharmacol* 69(3)**,** 825-834. doi: 10.1007/s00280-011-1770-1.

Tan, W., Yamazaki, S., Johnson, T.R., Wang, R., O'Gorman, M.T., Kirkovsky, L., et al. (2017). Effects of Renal Function on Crizotinib Pharmacokinetics: Dose Recommendations for Patients with ALK-Positive Non-Small Cell Lung Cancer. *Clin Drug Investig* 37(4)**,** 363-373. doi: 10.1007/s40261-016-0490-z.

LIST OF ABBREVIATIONS

- **NSCLC:** Non-small cell lung cancer
- **GSI:** Gamma-secretase inhibitor
- **DBZ:** dibenzazepine
- **BMS-906024:** Bristol-Myers Squibb 906024
- **ANOVA:** Analysis of variance
- **KRAS:** Kirsten Rat Sarcoma oncogene
- **BRAF:** B- Rapidly accelerated fibrosarcoma serine/threonine protein kinase
- **RET:** Rearranged during transfection receptor tyrosine kinase
- **MET:** proto-oncogene receptor tyrosine kinase also called hepatocyte growth factor receptor
- **FDA:** Food and Drug Administration
- **EGFR:** Epidermal growth factor receptor
- **ALK:** Anaplastic Lymphoma Receptor Tyrosine Kinase
- **EML4-ALK:** Fusion protein echinoderm microtubule-associated protein-like 4 and anaplastic lymphoma kinase receptor tyrosine kinase
- **ROS1:** Receptor tyrosine kinase from the insulin family ROS1 (v-ros UR2 sarcoma virus oncogene homolog 1 receptor tyrosine kinase)
- **PI3K/Akt:** Phosphoinositide 3-kinase/Akt serine/threonine protein kinase also known as Protein kinase B
- **PI3KCA:** Phosphoinositide-3-kinase, catalytic, alpha polypeptide
- **TP53:** Tumor protein 53
- **NRAS:** N- Rapidly Accelerated Fibrosarcoma
- **SMARCA4:** SWI/SNF-Related Matrix-Associated Actin-Dependent Regulator of Chromatin Subfamily A Member 4
- **CDKN2A:** Cyclin Dependent Kinase Inhibitor 2A
- **MAML:** Mastermind Like Transcriptional Coactivator
- **DNA-PKc:** DNA-dependent protein kinase, catalytic subunit
- **MYC:** V-Myc Avian Myelocytomatosis Viral Oncogene
- **STR:** Short tandem repeat
- **DMSO:** Dimethyl sulfoxide
- **PVDF:** polyvinylidene difluoride
- **MCTS:** Multicellular tumor spheroid
- **SSGD:** Specific spheroid growth delay
- **T4xSV:** Time to reach 4x the volume at the start of treatment
- **RT:** Radiotherapy
- **LZAP:** leucine zipper containing ARF-binding protein
- **MTT assay:** Multi-Table Tournament assay
- **EMT:** Epithelial-Mesenchymal transition

SUPPLEMENTARY METHODOLOGY

Quantitative PCR

H1299 and H460 spheroids were grown as described above, on day 7 they were treated with DMSO or BMS-906024 at a final concentration of 1µM. Sixty spheroids/condition were collected at day 2 post-treatment and total RNA was isolated using NucleoSpin RNA (Macherey-Nagel) according to the manufacturer's protocol. Single-strand cDNA was obtained using Iscript cDNA synthesis kit (BioRad) followed by SYBR-green-based reverse transcription quantitative PCR (RT-PCR) using SensiMix SYBR high-ROX kit (GC Biotech). mRNA expression was quantified for NOTCH target genes *HES1* (forward: 5'-AGGCGGACATTCTGGAAATG-3' and reverse 5'-CGGTACTTCCCCAGCACACTT-3') and *c-MYC* (forward: 5'-TCAAGAGGCGAACACACAAC-3' and reverse 5'-GGTCTTTTCATTGTTTTCCA-3'). *RPL13A* was used as housekeeping gene (forward: 5'-CCGGGTTGGCTGGAAGTACC -3' and reverse 5'- CTTCTCGGCCTGTTTCCGTAG -3'). Cycle threshold (Ct) values were analyzed with CFX Connect Real Time System (BioRad). Two independent experiments were conducted for each cell line and measurements were performed in triplicate.

Spheroid Clonogenics

H1299 cells (1000 cells/well) were seeded in a 96-multiwell Greiner plate coated with 50 µl autoclaved 1.5% agarose (Sigma-Aldrich) in serum free DMEM medium to allow multicellular spheroid formation as previously described (Friedrich *et al*., 2009). On the fourth day post-seeding, a minimum of 60 spheroids/condition were treated with 2.5nM Paclitaxel and/or 1 µM BMS-906024 and/or 2 Gy radiation. Drug treatment washout (50% replacement with DMEM w/o drug) was performed 72 h post drug treatment. BMS-906024 was refreshed after 72 h of treatment.After 7 days of treatment, spheroids were collected and disaggregated with trypsin for 1h in a warm (37° C) bath without shaking. The cell suspension was counted and different densities between 500-5000 cells/well were seeded in 6 cm culture dishes and incubated for 11 days at 37° C. Colonies with over 50 cells were counted. Plating efficiency = colonies counted after 11 days / cells seeded in the dish. Survival fraction = plating $\text{efficiency}_{\text{treatment}}$ / plating $\text{efficiency}_{\text{control}}$.

SUPPLEMENTARY FIGURE AND TABLE LEGENDS:

Figure S1: **NOTCH signaling is active in multicellular H1299 and H460 spheroids.** Multicellular NCI-H460 and NCI-H1299 spheroid cultures were treated with 1 µM BMS-906024. mRNA expression of NOTCH target genes c*MYC* and *HES1* was assessed by qPCR 2-days post DMSO/BMS-906024 treatment. Two independent experiments (1 and 2) were analyzed with three replicas/condition/experiment. Mean and standard error of the mean are plotted.

Figure S2: The addition of the pan-NOTCH γ-secretase inhibitor BMS-906024 to chemotherapy and to chemoradiation delays NCI-H460 multicellular spheroid specific growth. Multicellular NCI-H460 spheroids were treated with 2.5 nM Paclitaxel (A) or 0.8 µM Crizotinib (B) and/or 1 µM BMS-906024 and/or 2 Gy radiation. A minimum of 12 individual spheroids were tested per condition. *p-value<0.001 (***).*

Figure S3: Pan-NOTCH / gamma-secretase inhibitor BMS-906024 decreases clonogenic survival of Paclitaxel plus radiation regimen in NCI-H1299 multicellular spheroids. Clonogenic assay of H1299 spheroids treated with 2.5 nM Paclitaxel, with DMSO/1 µM BMS-906024, and 2 Gy radiation. Two independent experiments with three replicas / condition / experiment were done. Mean and standard error of the mean for the 500 cell density are plotted.

Figure S4: Best treatment combinations in NSCLC multicellular spheroids differ between treatment modalities. Comparison in multicellular NCI-H460 spheroids of: chemotherapy (1 nM or 2.5 nM paclitaxel, 0.4 µM or 0.8 µM crizotinib) versus chemotherapy plus 1 µM BMS-906024 (A), and chemoradiation (with 2 or 4 Gy) versus chemoradiation plus 1 µM BMS-906024 (B), is shown. **Red bars** indicate that the addition of BMS-906024 to that treatment option conferred a statistically significant SSGD compared to the treatment without BMS-906024, and the interaction was synergistic. **Green bars** indicate only statistically significant SSGD. **Blue bars** indicate only synergistic interaction. **Black bars** indicate non-significant relationship when NOTCH inhibitor is added.

Tables S1.1 and S1.2: Qualitative analysis of semi-high throughput screen of chemoradiation treatments on growth of NCI-H1299 cells in monolayer. Synergistic, protective, additive and toxic interactions between clinically approved compounds from the NCI/CTEP library at 0.77 µM with/without 4Gy radiation were determined on two biological replicates with two technical replicates per experiment.

Tables S2.1 and S2.2: Qualitative analysis of semi-high throughput screen of chemoradiation treatments on growth of NCI-H460 cells in monolayer. Synergistic, protective, additive and toxic interactions between clinically approved compounds from the NCI/CTEP library at 0.77 µM with/without 4Gy radiation were determined on two biological replicates with two technical replicates per experiment.

Table S3: Synergistic interactions and statistical significance between specific growth delays of newly suggested treatments including the pan-NOTCH / γ-secretase inhibitor BMS-906024 compared to first line treatment options for NSCLC in NCI-H1299 spheroids. Extended overview of Table 1.

Table S4: Synergistic interactions and statistical significance between SSGD of newly suggested treatments including the pan-NOTCH / γ-secretase inhibitor BMS-906024 compared to first line treatment options for NSCLC in NCI-H460 multicellular spheroids. Extended overview of Table 1.

Table S5: Reagent information
