# Supplementary material for: Synergistic Effects of NOTCH/γ-Secretase Inhibition and Standard of Care Treatment Modalities in Non-small Cell Lung Cancer Cells
Source: Front Oncol. 2018 Nov 7;8:460. doi: 10.3389/fonc.2018.00460 (PMC6234899; doi:10.3389/fonc.2018.00460)
Supplement: Supplementary file 2 [file Data_Sheet_2.pdf]

Supplementary Figure 1

A

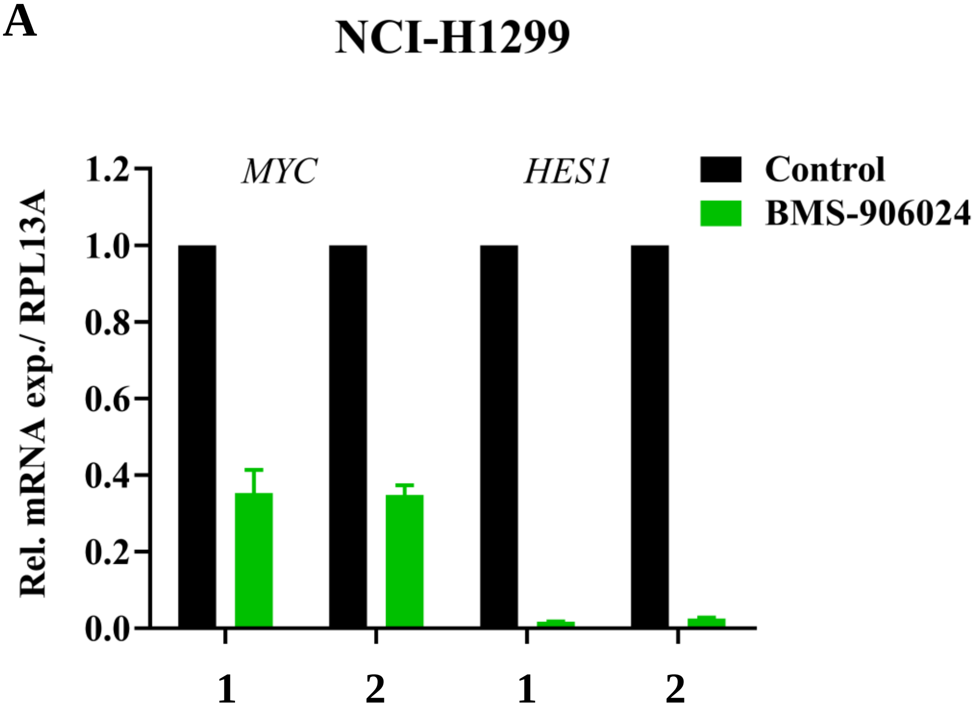

B

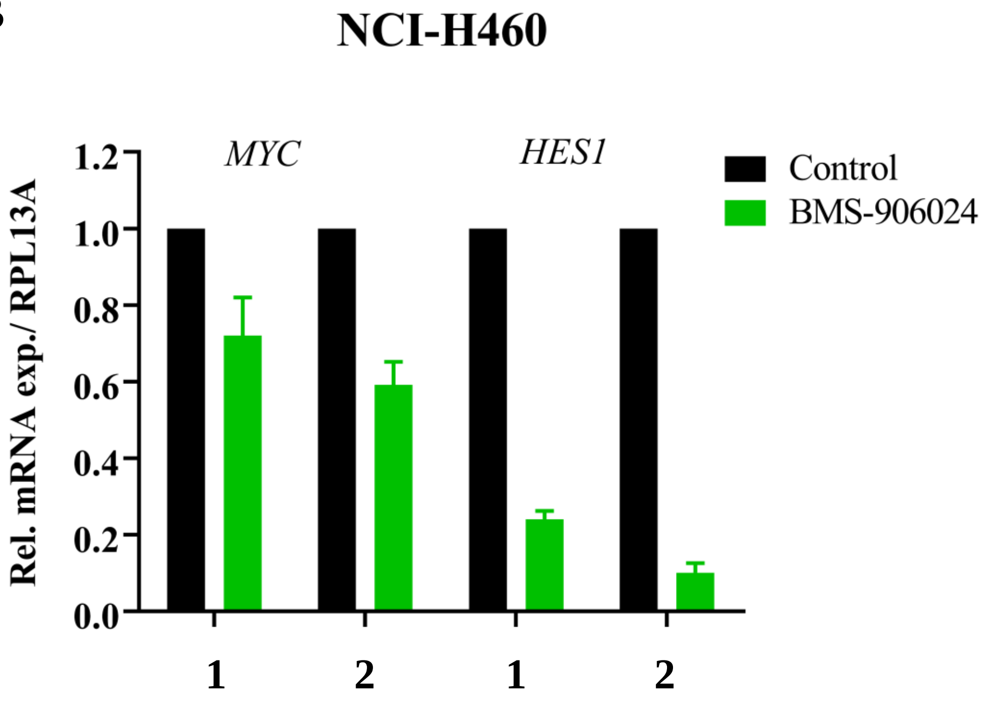

Supplementary Figure 2

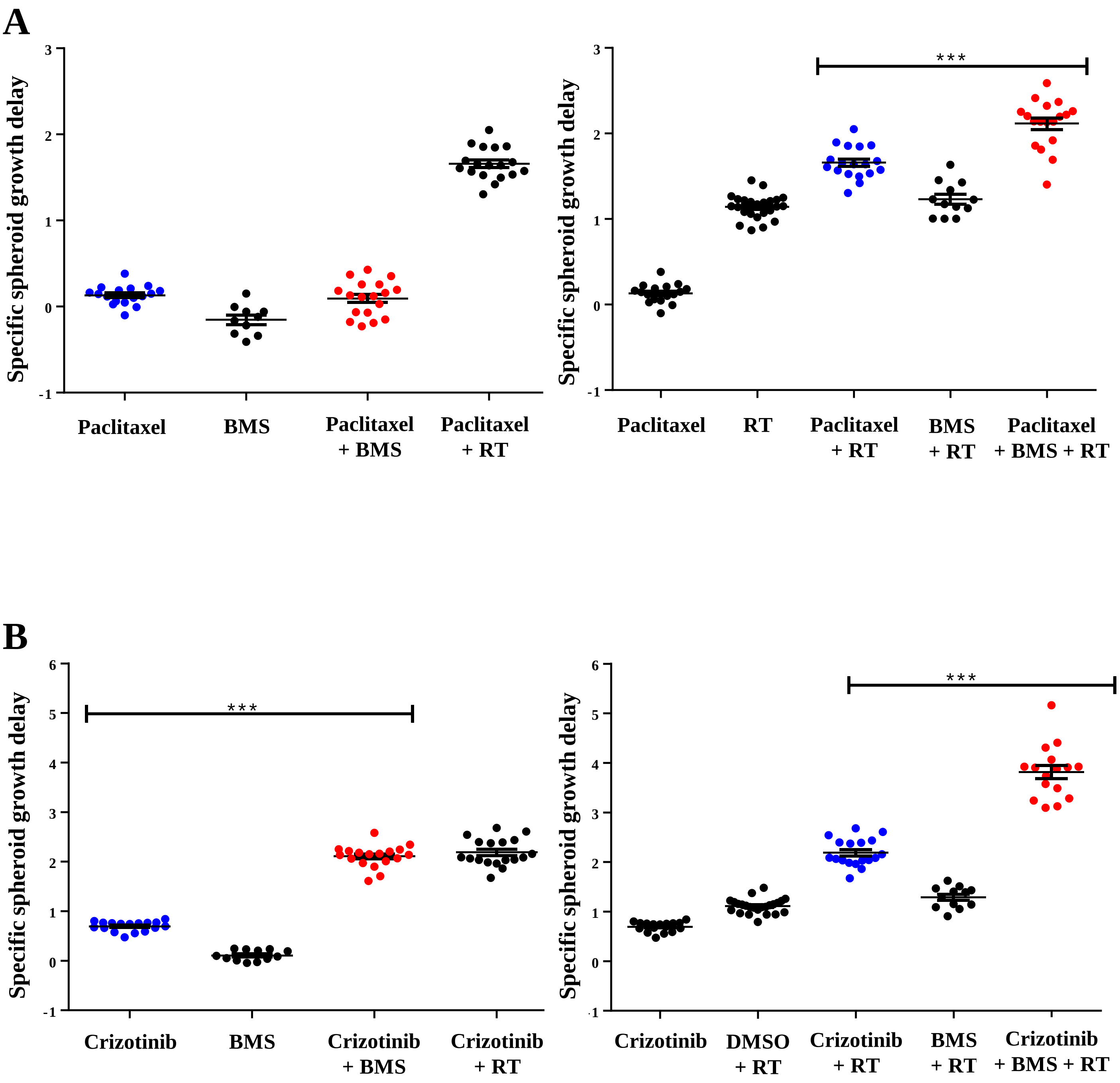

Supplementary Figure 3

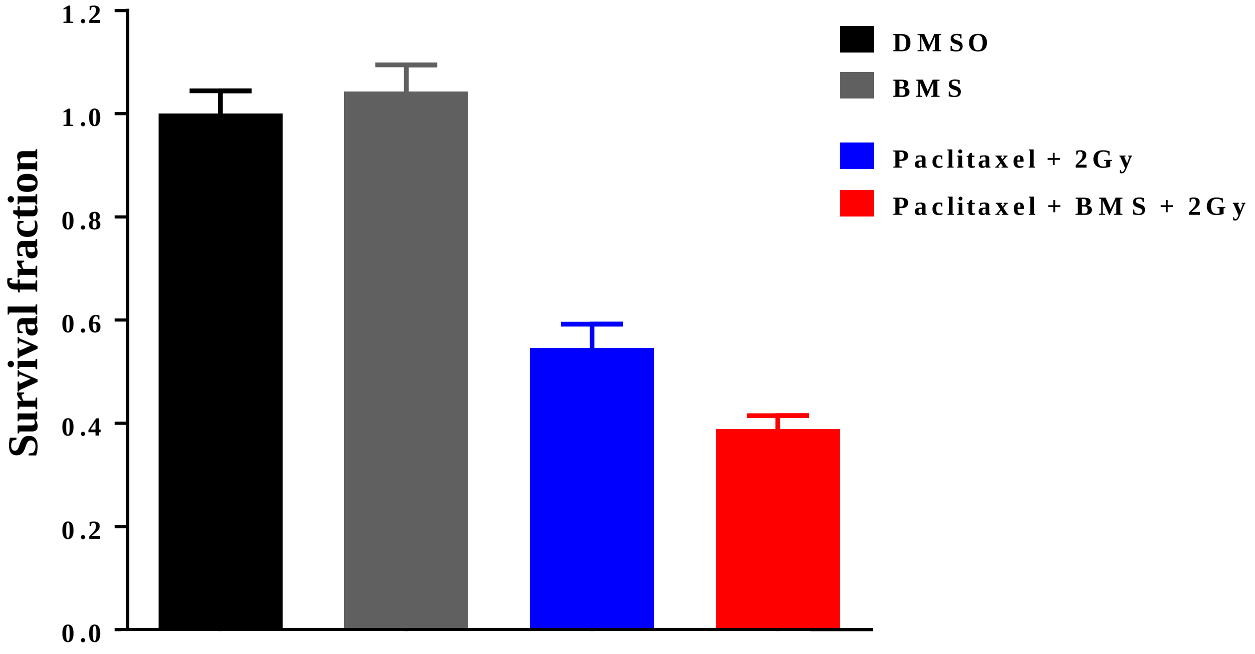

Supplementary Figure 4

A

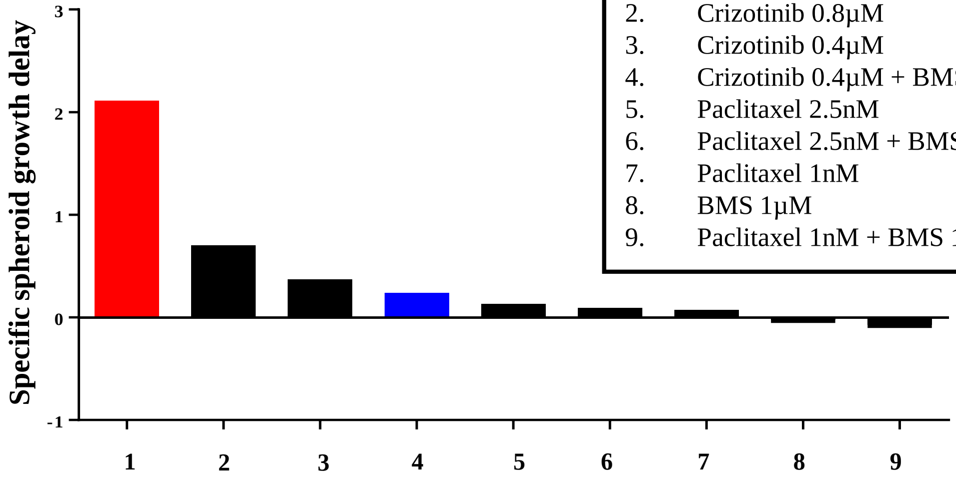

1. Crizotinib 0.8µM + BMS 1µM
2. Crizotinib 0.8µM
3. Crizotinib 0.4µM
4. Crizotinib 0.4µM + BMS 1µM
5. Paclitaxel 2.5nM
6. Paclitaxel 2.5nM + BMS 1µM
7. Paclitaxel 1nM
8. BMS 1µM
9. Paclitaxel 1nM + BMS 1µM

B

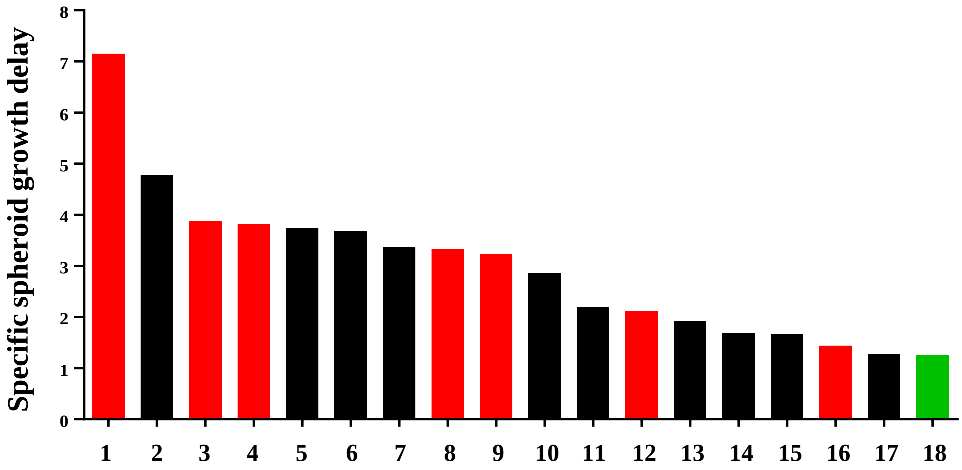

1. Crizotinib 0.8µM + BMS 1µM + 4Gy
2. Crizotinib 0.4µM + BMS 1µM + 4Gy
3. Paclitaxel 2.5nM + BMS 1µM + 4Gy
4. Crizotinib 0.8µM + BMS 1µM + 2Gy
5. Crizotinib 0.8µM + 4Gy
6. Crizotinib 0.4µM + 4Gy
7. Paclitaxel 2.5nM + 4Gy
8. Paclitaxel 1nM + BMS 1µM + 4Gy
9. BMS 1µM + 4Gy
10. Paclitaxel 1nM + 4Gy
11. Crizotinib 0.8µM + 2Gy
12. Paclitaxel 2.5nM + BMS 1µM + 2Gy
13. Crizotinib 0.4µM + BMS 1µM + 2Gy
14. Crizotinib 0.4µM + 2Gy
15. Paclitaxel 2.5nM + 2Gy
16. Paclitaxel 1nM + BMS 1µM + 2Gy
17. Paclitaxel 1nM + 2Gy
18. BMS 1µM + 2Gy

Supplementary Table 1.1

DRUG NAME	Synergistic	Protective	Additive	Toxic	No effects
Hydroxyurea					x
Allopurinol					x
Fluorouracil (5-FU)					x
Thioguanine					x
Mercaptopurine					x
Mechlorethamine HCl					x
Thiotepa	x				
Aminolevulinic Acid	x				
Dacarbazine	x				
Arsenic Trioxide	x				
Temozolomide					x
Busulfan					x
Altretamine	x				
Floxuridine				x	
Methoxsalen					x
Lomustine; CCNU					x
Azacitidine	x				
Decitabine			x		
Carmustine	x				
Cyclophosphamide					x
Uracil mustard			x		
Cytarabine; Ara-C				x	
Thalidomide					x
Procarbazine HCl		x			
Streptozocin	x				
Cladribine	x				
Ifosfamide					x
Cisplatin					x
Tretinoin					x
Dexrazoxane HCl			x		
Pentostatin		x			
Gemcitabine HCl					x
Nelarabine			x		
Vorinostat			x		
Exemestane			x		
Anastrozole					x
Letrozole	x				
Lenalidomide	x				
Chlorambucil		x			
Mitomycin C					x
Mitotane; o;p'-DDD			x		
Clofarabine				x	
Pipobroman			x		
Megestrol acetate	x				
Bendamustine HCl					x
Carboplatin			x		
Oxaliplatin		x			
Fludarabine Phosphate					x
Bortezomib				x	
Capecitabine	x				

Supplementary Table 1.2

DRUG NAME	Synergistic	Protective	Additive	Toxic	No effects
Celecoxib			x		
Sunitinib Malate			x		
Axitinib	x				
Mitoxantrone HCl				x	
Pemetrexed Disodium	x				
Gefitinib				x	
Vismodegib	x				
Crizotinib	x				
Methotrexate				x	
Quinacrine			x		
Topotecan HCl				x	
Dasatinib			x		
Pazopanib HCl					x
Imatinib Mesylate				x	
Sorafenib	x				
Raloxifene HCl			x		
Pralatrexate				x	
Vandetanib				x	
Vemurafenib				x	
Ixabepilone				x	
Romidepsin					x
Daunorubicin HCl					x
Doxorubicin HCl	x				
Etoposide	x				
Tamoxifen Citrate			x		
Lapatinib Ditosylate			x		
Irinotecan HCl			x		
Fulvestrant					x
Teniposide				x	
Valrubicin		x			
Docetaxel	x				
Cabazitaxel					x
Paclitaxel				x	
Vinblastine Sulfate				x	
Vincristine Sulfate					x
Sirolimus (Rapamycin)		x			
Everolimus				x	
Dactinomycin				x	
Plicamycin				x	
Bleomycin				x	
Vinorelbine Tartrate				x	
Carfilzomib				x	
Imiquimod				x	
Triethylenemelamine				x	
Erlotinib HCl				x	
Amifostine					x
Zoledronic Acid				x	
Abiraterone				x	
Melphalan				x	
Nilotinib				x	
Estramustine phosphate		x			

Supplementary Table 2.1

DRUG NAME	Synergistic	Protective	Additive	Toxic	No effects
Hydroxyurea					x
Allopurinol		x			
Fluorouracil (5-FU)		x			
Thioguanine					x
Mercaptopurine					x
Mechlorethamine HCl					x
Thiotepa	x				x
Aminolevulinic Acid					x
Dacarbazine					x
Arsenic Trioxide		x			
Temozolomide		x			
Busulfan					x
Altretamine					x
Floxuridine					x
Methoxsalen					x
Lomustine; CCNU					x
Azacitidine		x			
Decitabine		x			
Carmustine					x
Cyclophosphamide					x
Uracil mustard					x
Cytarabine; Ara-C				x	
Thalidomide					x
Procarbazine HCl					x
Streptozocin		x			
Cladribine					x
Ifosfamide		x			
Tretinoin		x			
Dexrazoxane HCl					x
Pentostatin					x
Gemcitabine HCl				x	
Nelarabine		x			
Vorinostat					x
Exemestane		x			
Anastrozole		x			
Letrozole					x
Lenalidomide					x
Chlorambucil					x
Mitomycin C				x	
Mitotane; o;p'-DDD			x		
Clofarabine	x				
Pipobroman					x
Megestrol acetate					x
Bendamustine HCl					x
Carboplatin					x
Oxaliplatin					x
Fludarabine Phosphate			x		x
Bortezomib				x	
Capecitabine					x

Supplementary Table 2.2

DRUG NAME	Synergistic	Protective	Additive	Toxic	No effects
Celecoxib					x
Sunitinib Malate					x
Axitinib					x
Mitoxantrone HCl					x
Pemetrexed Disodium			x		x
Gefitinib					x
Vismodegib					x
Crizotinib	x				
Methotrexate					x
Quinacrine					x
Topotecan HCl					x
Dasatinib			x		
Pazopanib HCl					x
Imatinib Mesylate					x
Sorafenib					x
Raloxifene HCl					x
Pralatrexate					x
Vandetanib					x
Vemurafenib					x
Ixabepilone					x
Romidepsin				x	x
Daunorubicin HCl				x	
Doxorubicin HCl			x		
Etoposide	x				
Tamoxifen Citrate					x
Lapatinib Ditosylate					x
Irinotecan HCl	x				
Fulvestrant		x			
Teniposide				x	
Valrubicin			x		x
Docetaxel			x	x	
Cabazitaxel					x
Paclitaxel				x	x
Vinblastine Sulfate				x	x
Vincristine Sulfate				x	
Sirolimus (Rapamycin)			x		
Everolimus			x		
Dactinomycin				x	
Plicamycin				x	
Bleomycin			x		
Vinorelbine Tartrate				x	
Carfilzomib				x	
Imiquimod					x
Triethylenemelamine			x		
Erlotinib HCl					x
Amifostine					x
Zoledronic Acid					x
Abiraterone					x
Melphalan					x
Nilotinib					x
Estramustine phosphate					x

Supplementary Table 3

H1299	Chemo Conc.	RT dose (Gy)	Specific spheroid growth delay difference			Synergism		
			Chemo vs Chemo + BMS	ChemoRT vs ChemoRT + BMS	BMS + RT vs ChemoRT + BMS	BMS vs Chemo	BMS vs ChemoRT	BMS vs RT
**Cisplatin χ**	**1.25 µM**	**2**	***	*	***	*ns*	**	*ns*
		**4**	**	***	***	*ns*	*ns*	*ns*
	**2.5 µM**	**2**	*ns*	***	***	**	*ns*	*ns*
		**4**	*ns*	*ns*	***	**	*ns*	*ns*
**Etoposide**	**0.1 µM**	**2**	***	**	*	*ns*	*ns*	*ns*
		**4**	***	*ns*	**	*ns*	*ns (0.0704)*	*ns*
	**0.25 µM**	**2**	*ns*	***	***	*	*ns*	*ns*
		**4**	*ns*	*ns*	***	*	*ns (0.0535)*	*ns*
**Paclitaxel**	**1 nM**	**2**	*ns*	*ns*	*ns*	*ns*	*ns*	*ns*
		**4**	*ns*	***	*ns*	*ns*	***	*ns*
	**2.5 nM**	**2**	*ns*	***	***	*ns*	***	*ns*
		**4**	*ns*	***	**	*ns*	***	*ns*
**Docetaxel**	**1 nM**	**2**	*** *(BMS effect)*	***	**	*	*	*ns*
		**4**	*** *(BMS effect)*	***	*ns*	*	*ns*	*ns*
**Pemetrexed χ**	**0.25 µM**	**2**	*ns*	*ns*	*ns*	*ns*	*ns*	*ns*
		**4**	*ns*	*ns*	*ns*	*ns*	*ns*	***
	**0.5 µM**	**2**	*ns*	*ns*	*ns*	*ns*	*ns*	*ns*
		**4**	*ns*	*ns*	*ns*	*ns*	*ns*	***
**Crizotinib**	**0.4 µM**	**2**	**	*	***	*ns*	*ns*	*ns*
	**0.8 µM**		*ns*	***	***	***	***	*ns*

Supplementary Table 4

H460	Chemo Conc.	RT dose (Gy)	Specific spheroid growth delay difference			Synergism		
			Chemo vs Chemo + BMS	ChemoRT vs ChemoRT + BMS	BMS + RT vs ChemoRT + BMS	BMS vs Chemo	BMS vs ChemoRT	BMS vs RT
Paclitaxel	1 nM	2	*ns*	*ns*	*	*ns*	***	***
		4	*ns*	**	*ns*	*ns*	**	***
	2.5 nM	2	*ns*	***	***	*ns*	***	***
		4	*ns*	*	***	*ns*	*	***
Crizotinib	0.4 µM	2	*ns*	**	***	***	*ns*	*ns*
		4	*ns*	***	***	***	*ns*	***
	0.8 µM	2	***	***	***	***	***	*ns*
		4	***	***	***	***	***	***

Supplementary Table 5

Product	Company	Cat. Number
DMEM High glucose with L-glutamine	Westburg	BE12-604F
RPMI 1640 with L-glutamine	Westburg	BE12-702F
Fetal Bovine serum	Sigma-Aldrich	F7524
Puromycin (solution) 100 mg	InvivoGen, Bio-Connect	ant-pr-1
Crizotinib	Selleckchem	s1068
DMSO	Sigma-Aldrich	41639
CELLSTAR ® 96-multiwell plates	Greiner	655180
CELLSTAR ® 12-multiwell plates	Greiner	665180
Bradford Protein Assay Dye reagent	Bio-Rad	5000006
Trizma ® base	Sigma-Aldrich	T6066-1KG
HCl	Sigma-Aldrich	H1758-500ml
NaCl	Sigma-Aldrich	S3014-1KG
Nonidet P-40	Affymetrix USB	19628
Sodium deoxycholate	Sigma-Aldrich	30970-25G
SDS	Bio-Rad	1610418
Sodium Fluoride	Sigma-Aldrich	S1504-500g
Sodium orthovanedate	Sigma-Aldrich	S6508-10g
Tween ® 20	VWR	8221841000
PVDF membrane	VWR	10600023
Anti-Cleaved NOTCH 1 Val1744 (D388) mAb rabbit	Bioke	4147S
Anti-Lamin A (C-terminal) rabbit	Sigma-Aldrich	L1293-200ul
Goat anti-rabbit IgG HRP-linked	Bioke	7074S
Amersham ECL™ prime WB detection reagent	Sigma-Aldrich	gerpn2232
Agarose for MCTS, Low electroendoosmosis	Sigma-Aldrich	A9539-100G
96-multiwell Ultra-Low attachment plates	Corning, BioScience	7007
